# Study on preoperative treatment of acute Type-A aortic dissection with endotracheal intubation combined with deep analgesia and sedation

**DOI:** 10.12669/pjms.40.1.8107

**Published:** 2024

**Authors:** Luo Chaoen, Asfandyar Khan, Hu Fan, He Shuangxi, Liu Qiaoling, Zhengwen Lei

**Affiliations:** 1 Luo Chaoen, Department of Thoracic and Cardiovascular Surgery, The First Affiliated Hospital, Hengyang Medical School, University of South China, Hengyang, Hunan, China; 2 Asfandyar Khan, Department of Thoracic and Cardiovascular Surgery, The First Affiliated Hospital, Hengyang Medical School, University of South China, Hengyang, Hunan, China; 3 Hu Fan, Department of Thoracic and Cardiovascular Surgery, The First Affiliated Hospital, Hengyang Medical School, University of South China, Hengyang, Hunan, China; 4 He Shuangxi Department of Thoracic and Cardiovascular Surgery, The Second Affiliated Hospital, Hengyang Medical School, University of South China, Hengyang, Hunan, China; 5 Liu Qiaoling Department of Anesthesiology, The First Affiliated Hospital, Hengyang Medical School, University of South China, Hengyang, Hunan,China; 6 Zhengwen Lei, Department of Thoracic and Cardiovascular Surgery, The First Affiliated Hospital, Hengyang Medical School, University of South China, Hengyang, Hunan, China

**Keywords:** Acute Stanford Type-A Aortic Dissection, Tracheal Intubation, Deep Analgesia and Sedation, Prevent Rupture

## Abstract

**Objectives:**

To investigate the efficacy and safety of endotracheal intubation combined with deep analgesia and sedation in the prevention of preoperative dissection rupture in acute Standford type A aortic dissection.

**Methods:**

This study evaluated the impact of preoperative endotracheal intubation combined with deep analgesia and sedation on acute Stanford Type-A aortic dissection. Conducted at the First Affiliated Hospital of the University of South China’s cardiac intensive care unit from June 2018 to December 2021, 134 diagnosed patients participated. They were divided into experimental (n=42) and control (n=92) groups. Data collected included clinical details, biochemical markers, VAS and SAS scores, and preoperative dissection rupture occurrences. Criteria involved acute Stanford Type-A aortic dissection diagnosis and complete data. Exclusions encompassed rupture, vital sign instability after vasoactive drugs, or prolonged coma. Standardized methods were used for sample collection and analysis. The study’s design, duration, and location ensured comprehensive evaluation of the intervention’s effects on patients.

**Results:**

The experimental group showed significantly fewer deaths due to dissection rupture compared to the control group (P < 0.05). Initial VAS and SAS scores (T0) were similar between groups (P > 0.05), indicating good comparability. However, at T1, T2, and T3, analgesia and sedation were significantly better in the experimental group (P < 0.05). By T4, patient numbers were too low in both groups for a significant difference (P > 0.05).

**Conclusion:**

Preoperative endotracheal intubation combined with deep analgesia and sedation in patients with acute Stanford Type-A aortic dissection can produce good analgesic and sedative effects, effectively reduce the incidence of preoperative dissection rupture, and create conditions for subsequent surgical treatment of patients.

## INTRODUCTION

Acute aortic type A dissection is a kind of cardiovascular disease with rapid onset, rapid progression, critical condition, high surgical mortality and postoperative complications. It is also one of the most difficult cardiovascular diseases in adult cardiovascular surgery. At present, the main treatment for acute Type-A aortic dissection is surgical treatment. The incidence of aortic Type-A dissection is approximately three to four per 10, 000 people world- wide,^1^ and in the absence of medical treatment, the mortality rate increases by 1% to 2% per hour after diagnosis,^2^ with more than 35% of patients dying within 24 hours of symptom onset and more than 50% within 48 hours. However, about 20% of patients lose the opportunity of surgery due to sudden death before surgery and cannot be treated.^3^ According to the recommendations in the Diagnosis and Treatment of Aortic Diseases of the European Society of Cardiology and the American College of Cardiology in 2014, medical drugs such as heart rate control, blood pressure control, defecation, absolute bed rest, analgesia and perfect preoperative preparation (class I B recommendation) plus emergency surgical treatment (class I C recommendation) were given.^4^ In clinical practice, however, even though we fully carried out in accordance with the guidelines of the recommended treatment for cough, roll over, but still can’t avoid patients’ urine, eat, vomiting, and pain, such as injection, on the way to the operating room, operating room bed stimulus and sudden large blood vessels to tear, bleeding, shock, and sudden death, and so on and so forth and lose the treatment opportunity.

Therefore, based on fully informing and communicating with family members of patients diagnosed with acute aortic Type-A dissection, our center plans to take the lead in adopting the method of endotracheal intubation + analgesia and sedation in China to put patients in the state of ventilator-assisted breathing + analgesia and sedation under general anesthesia to block all factors causing the increase of blood pressure of patients. Cardiovascular surgeons should enhance preoperative readiness promptly, schedule surgeries expeditiously, and enhance the surgical intervention rate for these patients. Simultaneously, there is a need to investigate the effectiveness and safety of utilizing endotracheal intubation in conjunction with analgesia and sedation to mitigate or diminish the occurrence of preoperative rupture in cases of acute Type-A aortic dissection.

## METHODS

### Grouping, inclusion, and exclusion criteria of cases

The grouping of cases took June 2018 as the time node, and patients with acute Stanford Type-A aortic dissection who did not receive endotracheal intubation combined with deep analgesia and sedation from June 2017 to August 2018 as the control group. Patients with acute Stanford Type-A aortic dissection who underwent endotracheal intubation combined with deep analgesia and sedation from June 2018 to December 2021 were included in the experimental group.

### Inclusion Criteria:

- Patients diagnosed as acute Stanford Type-A aortic dissection by aortic CTA after admission.
- Complete case data.
- The patient was conscious.

### Exclusion Criteria:

- Dissection rupture or failure to maintain vital signs after use of high-dose vasoactive drugs on admission.
- Coma has occurred for more than four hours.

### Clinical blood biochemistry test

After the patients with acute aortic Type-A dissection were admitted to the intensive care Unit of Cardiac Surgery, the First Affiliated Hospital of the University of South China, the nurses on duty in the intensive care unit of thoracic and cardiovascular surgery took 2ml of peripheral venous blood with a disposable venous blood sampling needle and placed it into a purple anticoagulant vacuum blood sampling tube. Two milliliter peripheral venous blood was collected and inserted into red vacuum blood collection vessel. Two milliliter peripheral venous blood was placed into blue vacuum blood collection vessel and sent to the laboratory of our hospital for examination. The automatic blood routine analyzer was used to test the samples in the purple anticoagulant vacuum blood collection tube, the automatic biochemical analyzer was used to test the samples in the red vacuum blood collection tube, and the automatic hemagglutination analyzer was used to test the samples in the blue vacuum blood collection tube. After verification, it is sent to the EMR electronic medical record system. Another 3ml of peripheral arterial blood was collected for blood gas analysis in our department. After verification, it is sent to the EMR electronic medical record system.

### Admission treatment

Basic Processing: In this study, all patients were admitted to the cardiac intensive care unit of our hospital after being diagnosed with acute Stanford Type-A aortic dissection. They were immediately given ECG monitoring and intensive care, and their respiration, consciousness, limb blood pressure, mental status, gastrointestinal tract and other conditions were comprehensively evaluated. To control heart rate, control blood pressure, analgesia, absolute bed and constipation and other symptomatic treatment. Two senior nurses on duty used VAS and SAS scores to evaluate the patients’ T0(immediately after admission to ICU), T1 (30 minutes of treatment), T2 (two hours of treatment), T3 (six hours of treatment), T4 (12 hours of treatment), to evaluate whether the patients’ analgesia and sedation reached the standard, and the control of blood pressure after analgesia and sedation. They also communicated with the patient’s family in detail, informed the patient’s condition and surgical plan, and signed the consent form for endotracheal intubation and central venipuncture.

### Catheterization of the right internal jugular vein

The right internal jugular vein was selected, and indwelling central venous catheter was placed after central vein puncture to establish venous access, and *β*-blockers and nitrate vasodilators were pumped according to the patient’s body weight.

### Preparation before endotracheal intubation + deep analgesia and sedation

Items and drug preparation: breathing machine, face mask, of laryngoscope and laryngoscope to size, trachea, sputum suction tube, infusion and injection pump, central venous catheter puncture suite, hydrogen morphine ketone, midazolam, sufentanil and propofol, vecuronium bromide, rui fentanyl (5% to 0.9% glucose or normal saline ratio of 50 ml).

### Administration sequence of endotracheal intubation plus deep analgesia and sedation

1. Hydromorphone 1mg (five minutes before intubation to reduce stress reaction before endotracheal intubation), pure oxygen for two minutes under mask, sufentanil 0.5-0.6ug/Kg midazolam 2-3mg, vicuronium 50mg/Kg propofol 1-2mg/kg. *Endotracheal intubation*:Sstomach satiation: Intubation was performed 30 seconds after vecuronium was pushed; Empty stomach: about 3-5 minutes after sufentanil takes effect, tracheal intubation should be performed. . *Analgesia and sedation*: About 5% glucose 50ml+ midazolam 20mg+ remifentanil 200mg and 5% glucose 50ml+ vecuronium 18mg were continuously pumped.

### Data Collection:

*Comparison of general clinical data between the experimental group and the control group:* The age, gender, smoking history, history of hypertension, history of diabetes, history of chronic obstructive pulmonary disease, history of Marfan syndrome, history of cardiovascular and cerebrovascular accident, and onset time were compared between the two groups.

### Comparison of blood biochemical indexes between experimental group and control group

Comparing the experimental group and control group before and after the two groups of endotracheal intubation + depth analgesia calm routine blood (white blood cells, neutrophils ratio), liver function (Alt, aspertate aminotransferase), renal function, creatinine, blood coagulation function (prothrombin time and the ratio of international standardization, blood gas, oxygen partial pressure, CO2 partial pressure, oxygen saturation, lactic acid value) is compared between groups.

### Observation Indicators

SBP values at T0, T1, T2, T3 and T4 were recorded. The number of patients with VAS score (0-3 points).Table-I and SAS score (2-4 points) (Table-II) in the experimental group and the control group were evaluated at T0, T1, T2, T3 and T4. And the numbers of dissection rupture before operation between the two groups were compared.

**Table-I:** Visual Analogue scale (VAS).

Score	The degree of pain
0	Painless
1-3	The patient had mild pain that was tolerable and did not interfere with sleep
4-6	The patient has moderate, tolerable pain that interferes with sleep
7-10	Patients with severe pain, unbearable, sweating, shortness of breath, affect sleep

***Note:*** A score of 0-3 indicates that the analgesic effect reaches the standard.

**Table-II:** Ricker (Sedation-Agitation Scale score).

Score	The degree of calm
1	No or only mild response to malignant stimuli, unable to communicate and follow instructions
2	Responsiveness to physical stimuli, inability to communicate and follow commands, voluntary
3	movement
4	Lethargy, verbal stimulation or gentle shaking can be awakened, and can follow simple commands
5	Quiet, easy to wake up, follows commands
6	Anxiety or agitation, medical words dissuade can be quiet
7	Very agitated, medicated, intubated, required protective restraint and repeated verbal discouragement Dangerous restlessness, trying to remove various catheters, attacking medical staff, tossing and turning in his hospital bed

***Note:*** A score of 2-4 indicates that the sedation effect reaches the standard.

### Statistical Analysi

Data were analyzed by SPSS25.0 software and GraphPad Prism software. Kolmogorov-Smirnov test was used to determine whether the distribution of variables was normal, and the quantitative indicators conforming to normal were statistically described by means (standard deviations), and the differences between groups were statistically inferred by t-test. Non-normal quantitative indicators were statistically inferred by Mann-Whitney U test and described by interquartile spacing. Qualitative data were described by frequency statistics, and differences between groups were statistically inferred by chi-square test.

### Ethical Considerations and Informed Consent

“In accordance with ethical guidelines and institutional regulations, this study was approved by the Institutional Review Board (IRB) of The First Affiliated Hospital of University of South China under protocol number 2022110214001. Written informed consent was obtained from all participants or their legal representatives prior to their inclusion in the study. The study was conducted in compliance with the principles outlined in the Declaration of Helsinki.”

## RESULTS

*:* There were no significant differences in age, gender, smoking history, hypertension history, diabetes history, COPD history, cerebrovascular history, Marfan syndrome and onset time between the two groups (P > 0.05). Table-III After admission, the patient was diagnosed with aortic Type-A dissection by aortic CTA, and the blood biochemical routine examination was completed immediately in emergency department. Blood biochemical test results of the two groups: White blood cells, ratio of neutral grain, cereal third transaminase, aspertate aminotransferase, creatinine, prothrombin time, ratio of international standardization, the partial pressure of oxygen partial pressure, carbon dioxide, oxygen saturation of comparison between groups, there was no statistically significant difference (P > 0.05), the experimental group lactic acid, lactic acid value is higher than the control group was statistically significant difference (P < 0.05), It may be related to the use of drugs (hydromorphone, midazolam, sufentanil, propofol, vecuronium, remifentanil) during endotracheal intubation, which causes transient hypotension and leads to relative hypoperperfusion of tissues and organs. Table-IV Comparison of sedation rate before endotracheal intubation combined with deep analgesia and sedation between two groups.

**Table-III:** Comparison of general data between the two groups

	The control group (n=92)	The experimental group (n=42)	Total (n=134)	T/x2	P value
Age /*χ̅*±*S*	56.11±11.65	54.36±12.26	55.56±11.82	-0.794	0.428
***Gender /n (%)***				3.667	0.056
Female	30 (32.6)	7 (16.7)	37 (27.6)		
Male	62 (67.4)	35 (83.3)	97(72.4)		
***Smoking history /n(%)***				2.619	0.155
Yes	35 (38.0)	10 (23.8)	45 (33.6)		
No	57 (62.0)	32 (76.2)	89 (66.2)		
***A history of high blood pressure /n (%)***				1.319	0.351
Yes	72 (78.3)	29 (69.0)	101 (75.4)		
No	20 (31.7)	13 (31.0)	33(24.3)		
***History of diabetes /n(%)***				0.927	0.336
Yes	2 (5.4)	0 (0.0)	2 (1.5)		
No	90 (94.6)	42 (100.0)	132(98.5)		
***COPD/n(%)***				2.371	0.124
Yes	5 (2.2)	0 (0.0)	2 (1.5)		
No	87 (97.8)	42 (100.0)	132(98.5)		
***History of cerebrovascular disease /n(%)***				0.150	0.699
Yes	5 (5.4)	3 (7.1)	8 (6.0)		
No	87 (94.6)	39 (92.9)	126(94.0)		
***Marfan syndrome/n(%)***				2.207	0.137
Yes	0 (0.0)	2 (4.8)	2 (1.5)		
No	92 (100.0)	40 (95.2)	132(98.5)		
***The onset of diseases/n (%)***				3.705	0.054
<8h	43 (47.3)	25 (59.5)	68 (51.1)		
8h-12h	12 (13.2)	10 (23.8)	22 (16.5)		
12h-24h	29 (31.9)	5 (11.9)	34 (25.6)		
>24h	7 (7.7)	2 (4.8)	9 (6.8)		

**Table-IV:** Comparison of blood biochemical indexes between the two groups before endotracheal intubation combined with deep analgesia and sedation.

Indexes	The control group (n=92)	The experimental group (n=42)	T/W	*P* value
WBC	12.96±4.33	14.37±4.43	-1.740	0.084
Neutrophil ratio	86.75 ±7.23	87.20±7.87	1691.500	0.249
Alanine aminotransferase	21.10 ±22.65	21.60±22.32	1828.000	0.618
Aspartate aminotransferase	27.05±27.81	30.50±39.60	1741.500	0.361
Creatinine	96.50±51.75	105.00±41	1628.500	0.145
Prothrombin time	12.10±1.7	12.15±2.63	1894.500	0.857
International normalized ratio	1.04±0.15	1.08±0.28	1702.000	0.270
Oxygen partial pressure	85.50±39.5	82.00±48.50	1874.000	0.781
Partial pressure of carbon dioxide	37.13±6.10	37.60±6.80	-0.398	0.691
Oxygen saturation	97.20±4.5	96.35±6.05	1872.000	0.773
Lactic acid *	1.60±1.8	2.30±4.16	1408.500	0.012

After admission, two senior nurses in the cardiac intensive care unit scored the VAS and SAS score system, in which VAS (0-3 points) indicated that analgesia reached the standard, and SAS (2-4 points) indicated that sedation reached the standard. Comparison between the experimental group and the control group: There was no significant difference in the proportion of VAS (0-3 points) and SAS (2-4 points) at T0 (P > 0.05), indicating that the experimental group was comparable with the control group. There were significant differences in the proportion of VAS (0-3 points) and SAS (2-4 points) during T0-T1 (P < 0.05). There were two deaths and one death in the control group and one death and zero death in the experimental group. There were significant differences in the proportion of VAS (0-3) and SAS (2-4) during T1-T2 (P < 0.05). During the period, four patients died in the control group, 17 patients were treated by surgery, and two patients died in the experimental group, eight patients were treated by surgery. There were significant differences in the proportion of VAS (0-3) and SAS (2-4) during T2-T3 (P < 0.05). There were eight deaths in the control group and 33 deaths in the operation group, while one death and 20 deaths in the experimental group. There was no significant difference in the proportion of VAS (0-3 points) and SAS (2-4 points) during T3-T4 (P > 0.05). During the period, 14 patients died in the control group and 10 patients were treated with surgery, while two patients died in the experimental group and seven patients were treated with surgery. Table-V. The number of dissection ruptures in the experimental group was lower than that in the control group, the difference was statistically significant (P < 0.05). Table-VI.

**Table-V:** Comparison of analgesia and sedation rate between the two groups before intubation.

	Indexes	The control group		The experimental group		X^2^	P value

		Yes	No	Yes	No		
T0	SAS (2-4)	12	80	11	31	3.505	0.061
	VAS (0-3)	10	82	7	35	0.875	0.350
T1	SAS (2-4)	59	30	40	1	15.113	0.000
	VAS (0-3)	66	23	41	0	12.873	0.000
T2	SAS (2-4)	35	33	30	1	19.381	0.000
	VAS (0-3)	37	31	31	0	20.575	0.000
T3	SAS (2-4)	12	15	10	0	9.343	0.002
	VAS (0-3)	15	12	10	0	6.578	0.010
	SAS (2-4)	2	1	1	0	0.444	0.505
	VAS (0-3)	2	1	1	0	0.444	0.505

***Note:*** All patients were planned to undergo emergency surgery after admission. However, due to different admission time, perfect preoperative preparation, and lack of blood source in some patients, patients received emergency surgery at different time periods after admission. Four patients in the experimental group were rescued immediately after admission and were given large doses of vasoactive drugs to maintain vital signs, but vital signs were still difficult to maintain, and died after rescue, accounting for 4.7% (two patients actually died before surgery).

**Table-VI:** Comparison of the number of dissection ruptures before operation between the experimental group and the control group.

Preoperative dissection rupture	Group		In total	X^2^	P value

	The experimental group (n=42)	Control group (n=92)			
Yes	6	29	35		
No	36	63	99	4.439	0.035
In total	42	92	134		

***Note:*** 42 patients after June 2018 were included in the experimental group (endotracheal intubation + deep analgesic sedation). In the control group, 29 patients died before operation, accounting for 31.5%. In the experimental group, 6 patients died before operation, accounting for 14.2%. Among them, 4 patients died after rescue with large dose of vasoactive drugs, accounting for 4.7% (two patients died before operation), but their vital signs could not be maintained.

## DISCUSSIONS

Acute Stanford Type-A aortic dissection is one of the common cardiovascular diseases in cardiovascular surgery. For most patients, once they are diagnosed with Stanford Type-A aortic dissection, timely surgical treatment is the best treatment plan at present. In recent years, with the progress of surgical technology, intensive care and anesthesia technology, the overall survival rate of Stanford Type-A aortic dissection has been significantly improved. However, the preoperative mortality rate is still very high^5^, and some patients die suddenly without timely surgical treatment after admission. In China, most tertiary hospitals lack the technology to perform surgical treatment for patients with Stanford Type-A aortic dissection. Therefore, it is necessary to transfer patients with Stanford Type-A aortic dissection to large cardiac and vascular surgical centers with surgical capabilities. However, during the transit, patients with Stanford Type-A aortic dissection may die due to various external stimuli and lose the opportunity for surgery. Similarly, even the safe transport to a large cardiac and vascular surgery center still faces many problems, among which the preparation of preoperative blood products is the most important. It has been reported in the literature that coagulation and fibrinolysis of Stanford Type-A aortic dissection are very significant before surgery, leading to a large amount of fibrinogen and platelet consumption.^6^ Therefore, A large amount of blood products, such as plasma, platelets, red blood cells, cryoprecipitate and other blood products, should be prepared before surgery for Stanford Type-A aortic dissection. Acute Stanford Type-A aortic dissection has rapid onset, dangerous condition, high surgical risk, and poor postoperative prognosis. In addition to the high cost of surgery, it takes time for families to face the risks. The interference of various external factors often prolongs the preparation time for surgery, which often increases the risk of preoperative death. How to reduce the preoperative mortality, prolong the operation preparation time, and improve the surgical treatment rate is the main purpose and core of this study.

Acute Stanford Type-A aortic dissection is A critical condition with high mortality. Therefore, once acute Stanford Type-A aortic dissection is diagnosed, admission to the intensive care unit is immediate. The patient’s condition and risk were evaluated, and the condition, risk and diagnosis and treatment plan were fully communicated with the family members in detail. After obtaining the consent of the family members and confirming the desire for surgery, the advantages and risks of preoperative endotracheal intubation plus deep analgesia and sedation and the follow-up treatment plan were informed as follows:

Make the patient in a state of anesthesia, reduce internal and external stimuli such as cough, turning over, defecation, pain, anxiety, fear, on the way to the operating room, and bed crossing in the operating room, so as to block the sharp increase in blood pressure caused by various stimuli, and reduce the risk of sudden death caused by dissection rupture and continuous tearing of great vessels.

The breathing machine accessory breathing + center venipuncture + continuous analgesia pump into sedation drugs, the patients achieve ideal effect of analgesia calm quickly, and can also prevent respiratory depression caused by the accumulation of opioids, promote analgesia sedative drugs together ho YiSiLuo hydrochloride and drugs in patients with better control of blood pressure, maintain a cycle stability.

The endotracheal intubation + depth analgesia after the calm, the related factors of causing increased blood pressure is blocked, in this golden time, we need to race against time perfect preoperative preparation for blood (frequent blood source nervous), blood type, improve the blood biochemical, electrocardiogram, bedside cardiac ultrasound, preoperative conversation, families and mutual assistance of blood donation and fund-raising and other work, Emergency surgical treatment was performed immediately after all preparations were completed. Since June 2018, our discipline and the Department of Anesthesiology have conducted regular multidisciplinary joint diagnosis and treatment, and formulated corresponding diagnosis and treatment plans, namely, endotracheal intubation plus deep analgesia and sedation for patients diagnosed with acute Stanford Type-A aortic dissection. In this study, a total of 134 patients were included and divided into two groups. Taking June 2018 as the time node, 92 patients before June 2018 were the control group (conventional treatment group), and 42 patients after June 2018 were the experimental group (endotracheal intubation + deep analgesia and sedation). As can be seen from Table-III, there was no statistically significant difference in the general data between the two groups (P > 0.05), and the two groups had good comparability. At present, there is A consensus that acute Stanford Type-A aortic dissection can cause systemic inflammatory reaction 7. Fan et al. believed that leukocytes and other inflammatory indicators were related to the in-hospital mortality of patients with dissection.^8^ As can be seen from Table-IV, there was no significant difference in inflammatory indicators (ratio of white blood cells and neutrophils) between the two groups (P > 0.05). Lactic acid, as the product of anaerobic digestion, is the product of poor systemic perfusion. Lactate level is an excellent surrogate for fatal sequelae of acute Stanford Type-A aortic dissection.^9^ Some studies have shown that lactate level is associated with short-term mortality after surgery.^10^ According to Table-IV, lactic acid in the experimental group was higher than that in the control group before intubation, and the difference was statistically significant (p < 0.05). The possible reasons for this are related to the relative hypoperfusion of organs and tissues caused by temporary decrease in blood pressure caused by narcotic drugs during intubation.^11^-^13^ We can get the following inspirations

 Endotracheal intubation combined with deep analgesia and sedation can reduce the related stress factors of patients and make the patient’s condition in a relatively stable state of analgesia and sedation. Meanwhile, most patients have already received surgical treatment before T4 stage (Table-V).

This method can give us a relatively valuable preoperative preparation time, so we need to improve the preoperative preparation as soon as possible and carry out surgical treatment. In the control group, 29 patients died before operation, accounting for 31.5%. Experimental preoperative death 6 people, accounted for 14.2%, including four patients admitted to hospital after began to rescue, high-dose asoactie drugs to maintain vital signs, but still difficult to maintain the vital signs, after the rescue invalid death, accounted for 4.7% in real terms, actual preoperative death, suggesting that preoperative to endotracheal intubation + depth analgesia calm can reduce the incidence of interlayer fracture, The difference was statistically significant (P < 0.05) . Table-VI. This method can win operation time for more patients.

For patients with acute Stanford Type-A aortic dissection, severe pain, combined with the lack of knowledge of the disease, the closed environment of ICU and the lack of company of relatives, can easily cause anxiety, anxiety, fear, restlessness and other psychological conditions, which is not conducive to the management of the disease,^14^ and even cause aggravation or deterioration of the disease. Endotracheal intubation refers to the insertion of an endotracheal tube through the mouth or nasal cavity through the larynx with the assistance of a laryngoscope, which is connected to a ventilator to establish an artificial airway to assist ventilation. Although simple endotracheal intubation (ventilator assisted breathing) can improve the oxygen and condition of acute Type-A aortic dissection, it may lead to restlessness, increased blood pressure, and even death of dissection rupture at any time when the patient is awake. Therefore, it is necessary to continuously pump analgesic and sedative drugs from the central venous catheter to keep the patient under complete anesthesia, that is, completely block the influence of pain, cough, turning over, defecation, eating, vomiting, anxiety, fear and other stimuli on blood pressure, and maintain a relatively stable hemodynamic state. In acute Stanford Type-A aortic dissection admitted to our Center, multidisciplinary assistance is often organized immediately to make treatment plans. Endotracheal intubation plus deep analgesia and sedation were performed under the guidance of anesthesiologists, and hydromorphone, midazolam, remifentanil, vecuronium and other drugs were administered according to the previous anesthesia induction and analgesic sedation protocol. Some studies have shown that hydromorphone can reduce the levels of adrenocorticotropic hormone and cortisol and reduce the stress response caused by pain.^15^ Low-dose midazolam + propofol + remifentanil + vecuronium can obtain good depth of anesthesia and reduce stress response by controlling blood pressure.^16^ Therefore, the risk of preoperative death caused by increased blood pressure and heart rate caused by tracheal intubation stimulation can be reduced. At present, there is no patient death caused by tracheal intubation in our center. After successful endotracheal intubation under anesthesia induction, midazolam + remifentanil and vecuronium were continuously pumped from the central vein according to the patient’s body weight and body surface area for maintenance of deep analgesia and sedation. As a sedative commonly used by anesthesiologists, midazolam’s effect on circulation changes with the dose change, and it has a capping effect. It also has the advantages of strong sedation, rapid onset and high safety, and can improve the incidence of delirium in patients with aortic dissection.^17^ Vecuronium is an amino steroid non-depolarizing muscle relaxant, which has almost no effect on the cardiovascular system, and intravenous anesthetics have no obvious effect on the muscle relaxant effect of vecuronium.^18^ Remifentanil is a highly effective opioid with the fastest onset (about one minute) and a short elimination half-life of 10 minutes. Remifentanil does not undergo liver and kidney metabolism and has good analgesic and sedative effects. At the same time, there is no need for anesthesia induction, endotracheal intubation and central venous catheterization after patients are admitted to the operating room, which saves anesthesia time and is conducive to emergency surgery as soon as possible.

Studies have shown that preoperative active medical conservative treatment should focus on reducing the excessive shear stress of diseased vessels in aortic dissection, so as to control the progress of the disease and prevent the occurrence of preoperative death.^19^ In this study, both the experimental group and the control group were routinely given Esmolol hydrochloride to control heart rate and reduce cardiac contractile force, and nitrates to control blood pressure. On this basis, the experimental group was given endotracheal intubation combined with deep analgesia and sedation, which kept the condition in a stable state and had a relatively low incidence of preoperative rupture. Therefore, simply focusing on the control of heart rate and blood pressure, one-sided sedation and analgesia, preoperative non-endotracheal intubation patients, and cannot be complete analgesia and sedation, often pain, anxiety, vomiting and other adverse stress factors are not well controlled, resulting in sudden malignant cardiac events. Endotracheal intubation combined with deep analgesia and sedation plus control of heart rate and blood pressure is helpful to maintain hemodynamic stability, strive to improve preoperative preparation as soon as possible, and improve the surgical treatment rate of acute Stanford Type-A aortic dissection, which is the core of this study.

In the study on preoperative treatment of acute Type-A aortic dissection with endotracheal intubation combined with deep analgesia and sedation, several factors have been investigated in relation to the prognosis and outcomes of patients. Two relevant studies have examined the association between specific parameters and patient outcomes in aortic dissection.

Sari et al. conducted a study to evaluate the prognostic significance of diastolic blood pressure in aortic dissection.^20^ They found that checking diastolic blood pressure twice is associated with prognosis. This finding suggests that monitoring diastolic blood pressure may provide important information for assessing the severity of aortic dissection and predicting outcomes.

Furthermore, Liu et al. investigated the impact of admission serum total cholesterol level on in-hospital mortality in patients with acute aortic dissection.^21^ Their study revealed that serum total cholesterol level at admission may be a relevant prognostic factor. This suggests that cholesterol levels could potentially serve as a marker to assess the severity of aortic dissection and predict the risk of mortality.

Taken together, these studies provide valuable insights into factors that may influence the prognosis of acute Type-A aortic dissection. Monitoring diastolic blood pressure and considering admission serum total cholesterol levels could potentially aid in risk stratification and guide the preoperative management of patients with aortic dissection. However, further research is needed to validate and explore the clinical implications of these findings in the context of preoperative treatment strategies involving endotracheal intubation combined with deep analgesia and sedation.

### Significance of this study

The significance of this study lies in its novel approach to improving preoperative care for individuals diagnosed with acute Stanford Type-A aortic dissection. By using a combination of methods, including endotracheal intubation and deep analgesia, and sedation, the research aimed to establish a stable physiological state that reduces the risk of preoperative dissection rupture and improves the chances of successful surgical treatment. The study’s findings hold important implications. Firstly, the intervention demonstrated a noticeable decrease in preoperative mortality by preventing sudden cardiac events caused by stress-related factors and uncontrolled blood pressure. Secondly, the approach provided valuable time for thorough preoperative preparation, crucial for patients in critical conditions. This preparation time can contribute to better surgical outcomes and increase the overall success rate of treatments. Furthermore, the results indicated that patients who underwent the combined technique experienced improved outcomes and a higher rate of successful surgical interventions. As a result, this strategy can enable surgical teams to promptly and effectively perform interventions when necessary. Ultimately, this study could have a positive impact on clinical practices in cardiovascular surgery, introducing a new method to address the challenges associated with managing acute Stanford Type-A aortic dissection. The integration of endotracheal intubation and deep analgesia and sedation offers a promising avenue for improved patient outcomes, reduced mortality rates, and an increased likelihood of successful surgical treatments.

### Limitations

This study is a single-center study, and the number of included cases is small, so the sample size needs to be expanded. Statistical analysis of postoperative conditions has not been performed. The follow-up research is planned to explore the regulatory mechanism of related stress response or inflammatory factors from the perspective of combining basic experiments and clinical research, taking blood and tissue samples of patients as the entry point.

## CONCLUSIONS

1. Endotracheal intubation combined with deep analgesia and sedation can produce good analgesic and sedative effects, effectively reduce the incidence of preoperative death in patients with acute Stanford Type-A aortic dissection and create opportunities for surgical treatment of patients.
2. Endotracheal intubation combined with deep analgesia and sedation can provide surgeons with relatively precious preoperative preparation time, fully improve emergency surgical treatment after preoperative preparation, and enhance the success rate of surgical interventions.
3. Complete preoperative endotracheal intubation + deep analgesia and sedation, patients can be safely and smoothly sent to the operating room, and save the time of anesthesia induction, endotracheal intubation, and central venous catheterization, which is conducive to emergency surgery as soon as possible.

### Authors Contribution

**LC** conceived, designed and did the data collection.

**AK** did data collection, analysis and interpretation of data.

**HF** contributed in data collection.

**HS** did the manuscript writing.

**LQ, ZL** Conceived and revised the manuscript.

**ZL** takes the responsibility and is accountable for all aspects of the work in ensuring that questions related to the accuracy or integrity of any part of the work are appropriately investigated and resolved.
